# Sensitivity and specificity of the Major Depression Inventory in outpatients

**DOI:** 10.1186/1471-244X-7-39

**Published:** 2007-08-09

**Authors:** Pim Cuijpers, Jack Dekker, Annemieke Noteboom, Niels Smits, Jaap Peen

**Affiliations:** 1 Department of Clinical Psychology, VU University Amsterdam, Amsterdam, The Netherlands; 2 Mentrum Mental Health Care, Amsterdam, The Netherlands

## Abstract

**Background:**

The Major Depression Inventory (MDI) is a new, brief, self-report measure for depression based on the DSM-system, which allows clinicians to assess the presence of a depressive disorder according to the DSM-IV, but also to assess the severity of the depressive symptoms.

**Methods:**

We examined the sensitivity, specificity, and psychometric qualities of the MDI in a consecutive sample of 258 psychiatric outpatients. Of these patients, 120 had a mood disorder (70 major depression, 49 dysthymia). A total of 139 subjects had a comorbid axis-I diagnosis, and 91 subjects had a comorbid personality disorder.

**Results:**

Crohnbach's alpha of the MDI was a satisfactory 0.89, and the correlation between the MDI and the depression subscale of the SCL-90 was 0.79 (p < .001). Subjects with major depressive disorder (MDD) had a significantly higher MDI score than subjects with anxiety disorders (but no MDD), dysthymias, bipolar, psychotic, other neurotic disorders, and subjects with relational problems. In ROC analysis we found that the area under the curve was 0.68 for the MDI. A good cut-off point for the MDI seems to be 26, with a sensitivity of 0.66, and a specificity of 0.63. The indication of the presence of MDD based on the MDI had a moderate agreement with the diagnosis made by a psychiatrist (kappa: 0.26).

**Conclusion:**

The MDI is an attractive, brief depression inventory, which seems to be a reliable tool for assessing depression in psychiatric outpatients.

## Background

Most rating scales which measure the presence and severity of depressive symptoms were developed before the release of the evidence-based diagnostic system DSM-III in 1980 [1,2]. Therefore, the items of these scales are not based on the DSM system, and may not be the optimal way of measuring major depression. One way to solve this problem is to modify the older instruments and adapt to the modern concept of depressive disorder, as has been done with the 40-year old Hamilton Depression Scale (HAM-D) [3], and the equally old BDI [4,5]. Another way to solve this problem is by developing new instruments, which cover DSM and ICD items better. Several of these instruments have been developed in the past years, such as the IDS and QIDS [6], and the PHQ-9 [7].

One other self-report measure for depression which is based on the DSM-system is the Major Depression Inventory (MDI). The MDI includes all symptoms of depression in the DSM-IV and the ICD-10. Each of these symptoms is rated on a six-point scale, which allows clinicians to assess the presence of a depressive disorder according to the DSM-IV and the ICD-10 (each symptom is dichotomized), but also to assess the severity of the depressive symptoms (by summing up the scores of all symptoms, with a range of 0 to 50). The MDI is attractive because it is brief, it allows clinicians to assess the presence of a depressive disorder, and it can be filled in by the patient himself.

Research examining the psychometric qualities of the MDI is limited. Earlier studies have evaluated the applicability and internal validity of the MDI [8], the sensitivity and specificity in psychiatric outpatients [2], and the psychometric properties in general [9]. The results of these studies indicate that the MDI has good sensitivity, specificity, reliability and validity. Because the number of studies is limited and because these studies are conducted in relatively small samples, more research is needed to confirm the positive results of earlier research. Furthermore, for diagnostic classification, studies with mixed groups of outpatients are needed to confirm the validity and applicability of the scale.

In the current study, we will examine the sensitivity, specificity, and psychometric qualities of the MDI in a naturalistic investigation including 258 outpatients in mental health care.

## Methods

### Subjects and procedure

Subjects were patients who sought help for mental disorders at a large outpatient clinic ("Roeterstraat") of Mentrum Mental Health Care in Amsterdam. Data were collected in a routine intake procedure of this outpatient clinic, in which patients were asked to fill in several instruments for the purpose of diagnostic assessment of the personality and therapy indication. Conforming to current legal requirements and with approval of a medical ethical committee (full name of the committee is: "METIGG; Stichting Medisch-Ethische Toetsingscommissie Instellingen Geestelijke Gezondheidszorg"), patients were informed that these anonimised routine clinical data may be used for the purpose of a scientific assessment study. If the patient did not want that their data were used for research purposes, the patient could report this to the research coordinator and in such a case the data were not used.

Mental disorders were assessed according to the DSM-IV criteria by six experienced psychiatrists in a regular diagnostic interview. These psychiatrists had three-weekly consensus meetings during which they discussed the diagnoses of the included patients. In keeping with current Dutch legal requirements, patients were informed that anonimised routine clinical data might be used for the purpose of research and given the choice to "opt out", in which case their data were not used.

A consecutive sample of 465 subjects was used for this study. Of these 465 subjects, 99 were not assigned a diagnosis during the intake phase (because they did not show up at their first appointment at the outpatient clinic, or dropped out during the intake phase) and were removed from the dataset. Of the remaining 366 subjects, 108 did not fill in the MDI (N = 90) or not completely (N = 18), mainly because of language problems. Of the remaining 258 subjects with an MDI, 208 had an SCL-D score, and 229 had an SCL-A score.

Selected characteristics of the resulting sample of 258 subjects are presented in Table 1. Almost half of the subjects were male, most had a higher education, and were younger than 40 years of age.

**Table 1:** Selected characteristics of the included sample outpatients in mental health care (N = 258)

		N	%
Gender	Male	111	43.0
	Female	142	55.0
	Missing	5	1.9
Education	Lower	16	6.2
	Higher	106	41.1
	Unknown	136	52.7
Age	< 40 years	171	66.3
	≥ 40 years	86	33.3
	Missing	1	0.4
	M (SD)	36.45	(10.13)
			
Primary diagnosis	Mood disorder	102	39.5
	- MDD	63	24.4
	- Dysthymia	33	12.8
	- Other mood^a)^	6	2.3
	Bipolar disorder	10	3.9
	Anxiety disorder	41	15.9
	Psychotic disorder	12	4.7
	Other neurotic disorder	36	36
	Relational problems	25	9.7
	Substance use disorder	16	6.2
	Other^b)^	16	6.2

^a) ^depressive disorder caused by a somatic illness (N = 1), or a depressive disorder Not Otherwise Specified (N = 7).

^b) ^other disorders are specified in the text

The following Axis-I-disorders were found: depressive disorders (major depression (293.83, 296.2x, 296.3x), dysthymia (300.4) and depression Not Otherwise Specified (311, 296.90)), anxiety disorders (phobic disorders, panic disorder, generalized anxiety and obsessive-compulsive disorder (300.0x, 300.1, 300.2x, 308.3, 309.81)), bipolar disorder (296.0x, 296.4x, 296.5x, 296.6x, 296.7, 296.8x, 301.13), schizophrenia and other psychotic disorders (293, 293.81, 293.82, 295.xx, 297.xx, 298.xx), other neurotic disorders (300.11–15, 300.6, 300.7, 300.81, 307.8x, 309.0, 309.24, 309.28, 309.3, 309.4, 309.9), relational problems (V61.xx, V62.xx, V71.01, V71.02, V15.81, V65.2, 780.9, 313.82), substance use disorder (291.xx, 292.xx, 303.xx, 305.xx) and other disorders (all the other codes grouped).

As can be seen in Table 1, most subjects had a mood disorder, anxiety disorder, or another neurotic disorder as the primary DSM-IV diagnosis. Apart from the primary diagnosis, 139 subjects had a secondary DSM-IV axis-I diagnosis. A total of 91 subjects also had a personality disorder.

One hundred and twenty subjects (46.5%) had a mood disorder (as the first or second diagnosis; Table 2). Of these 120 subjects, 70 had a major depressive disorder, while 49 had dysthymia. Eight subjects had another depressive disorder (in one case caused by somatic illness, in the other cases it was a depressive disorder Not Otherwise Specified).

**Table 2:** Means and standard deviation at the MDI in psychiatric outpatients

	All			Men			Women		
			
	N	M	SD^c)^	N	M	SD^c)^	N	M	SD^c)^
Mood disorder	120	27.69	10.60 ***	54	27.57	9.46 **	63	27.33	11.54 **
- MDD	70	29.96	9.97 ***	29	29.10	8.89 **	40	30.30	10.75 ***
- Dysthymia	49	25.80	11.65	24	25.08	10.62	22	25.00	12.75
- Other mood^a)^	8	21.88	8.87	3	25.00	4.58	5	20.00	10.75
Bipolar disorder	11	15.64	11.84 *	9	11.33	7.73 ***	2	35.00	2.83
Anxiety disorder	59	24.32	11.84	23	28.61	9.16 *	35	22.03	12.53
Psychotic disorder	12	19.50	11.81	10	21.10	12.20	2	11.50	6.36
Other neurotic disorder	43	23.33	11.86	15	23.33	8.80	27	23.26	13.62
Relational problems	62	21.11	12.31 *	21	19.52	11.47 *	41	21.93	12.78
Substance use disorder	44	26.52	11.84	30	25.57	11.79	13	27.62	12.06
Other^b)^	26	21.35	12.94	7	25.57	12.90	19	19.79	12.95
									
Total population	258	24.03	12.12	111	23.99	11.17	142	23.91	12.79

***: p < .001; **: p < .01; *: p < .05; ns: not significant

^a) ^depressive disorder caused by a somatic illness (N = 1), or a depressive disorder Not Otherwise Specified (N = 7).

^b) ^other disorders are specified in the text

^c) ^p-values indicate whether the mean MDI-scores in the diagnostic category differ significantly from the rest of the population, as tested with T-tests.

### Measures

Major Depression Inventory (MDI). The MDI is a 12-item self-report questionnaire for depression, intended to measure DSM-IV diagnosis of major depression by the patients' self-reported symptoms. The items of the MDI ask the patient to rate how much time in the past two weeks each of the symptoms of the depressive syndrome was present (on a six-point rating scale ranging from none to all of the time). As indicated earlier, the MDI can be used to get an indication of the presence of major depression (according to the algorithm of the DSM-IV), or as an instrument measuring severity of depression (with a range of 0 to 60).

We used the Dutch translation of the MDI. The translation and back translation of the MDI was made by two of the authors (PC and JD); one of whom did the translation and the other who did not know the original English text, did the back translation. The final translation was fixed by consensus.

Symptom Checklist (SCL-90) – depression subscale and anxiety subscale. Depressive symptomatology was also assessed with the Depression Scale of the Symptom Check List – 90, Dutch version [10]. This self-report scale measures the severity of symptoms and consists of 16 psychological symptoms which must be rated on a 5-point scale, ranging from 1 (not distressed by the symptom) to 5 (extremely distressed by the symptom). A total score can be obtained by adding the item scores, ranging from 16 (no depressive symptomatology) to 80 (high level of depressive symptoms). The anxiety subscale of the SCL-90 consists of 10 items indicating the degree to which a person had symptoms of anxiety in the past week (range 10 to 50). The SCL-90 is a much used instrument with excellent psychometric properties [10].

DSM-IV diagnoses. As indicated earlier, all mental disorders of patients were assessed according to the DSM-IV criteria by experienced psychiatrists in a regular diagnostic interview.

### Analyses

In the first part of the analyses, we examined the MDI as a measure indicating the severity of the depressive symptomatology.

First, we calculated the reliability of the MDI (Cronbachs alpha). We also calculated the correlation with the SCL-90 depression subscale. A high correlation supports the construct validity of the MDI.

Then we calculated the means and standard deviations (SDs) of the MDI in all diagnostic categories (major depression; dysthymia; anxiety disorder; psychotic disorder; and other). We furthermore calculated means and SDs separately for men and women. We subsequently examined with *t*-tests whether the mean MDI score differed in subjects with major depression compared to subjects with other diagnoses. Significantly higher MDI-scores in subjects with major depression can be seen as an indication that the MDI actually is a measure of depression.

We also calculated the sensitivity and specificity of different cut-off values at the MDI in detecting major depression and dysthymia, and performed Receiver Operating Characteristic (ROC) analyses. We also performed ROC analyses for the SCL-90-D, and we tested the equality of the MDI and the SCL-90-D.

In the second part of the analyses, we concentrated on the MDI as an instrument which gives an indication of the presence of MDD, and compared it to the MDD diagnosis as given by the psychiatrist. We calculated the sensitivity and specificity of the MDI-indication of a DSM-IV diagnosis of major depression, in different subpopulations. In these analyses, we calculated the kappa statistic as an indication of the agreement between the judgment of the psychiatrist and the MDI.

The *t*-tests, reliability analyses, correlations, and the calculations of the sensitivity, specificity, and kappa statistic were conducted in SPSS 12.0.02; the ROC analyses were conducted in STATA/SE 8.2 (which permits testing of the equality of two different measures given a gold standard).

## Results

### The MDI as a measure of severity

The reliability of the MDI, as indicated with Crohnbachs alpha was a satisfactory 0.89.

The correlation between the scores of the MDI and the depression subscale of the SCL was 0.79 (p < .001), and between the MDI and the anxiety subscale of the SCL 0.57 (p < .001).

The means and SD on the MDI for the different diagnostic categories are presented in Table 2. Subjects with a mood disorder had a significantly higher MDI-score than the rest of our sample (p < .001), and this was true for men (p < .001) and for women (p < .01). When we differentiated among the subjects with a mood disorder, we found that subjects with MDD had a significantly increased MDI-score (p < .001), but subjects with dysthymia or another depressive disorder did not have a significantly increased score. Subjects with a bipolar disorder and those with relational problems had a significantly lower MDI score than the other subjects (p < .05). Men with a bipolar disorder (p < .001) or relational problems had a lower MDI score than other subjects (p < .05), while men with an anxiety disorder had an increased MDI score (p < .05).

Then we compared the mean MDI score in subjects with a major depressive disorder (but no anxiety disorder) to the MDI in subjects with an anxiety disorder (but not a comorbid major depressive disorder). Those with MDD had a significantly higher MDI score (M = 29.64; SD = 10.63; N = 59) than those with an anxiety disorder (M = 22.65; SD = 12.31; N = 48; p < .01)). In the same way, we compared subjects with MDD to subjects with dysthymia, with another depressive disorder, with a bipolar disorder, with a psychotic disorder, with another neurotic disorder, with relational problems, and with a substance use disorder. The subjects with dysthymia (M = 24.76; SD = 11.08; N = 41; p < .05) differed significantly from those with MDD (p < .01), as did those with another depressive disorder (M = 21.89; SD = 8.87; N = 8; p < .05), a bipolar disorder (M = 15.64; SD = 11.84; N = 11; p < .001), a psychotic disorder (M = 19.50; SD = 11.81; N = 12; p < .01), another neurotic disorder (M = 23.35; SD = 12.14; N = 40; p < .01), and those with relational problems (M = 19.28; SD = 11.63; N = 54; p < .001). There was a trend indicating a difference between the MDI score in those with MDD and those with a substance use disorder (M = 25.03; SD = 11.88; N = 38; p < .1).

Among the subjects with MDD, we examined whether there was a relationship between the MDI-score and demographic variables (gender, age, and education). We found no association between the MDI-score on the one hand, and gender and education on the other hand. However, we did find a significant correlation between MDI and age (r = 0.24; p < 0.05), indicating a somewhat higher MDI with increasing age.

**Figure 1:**
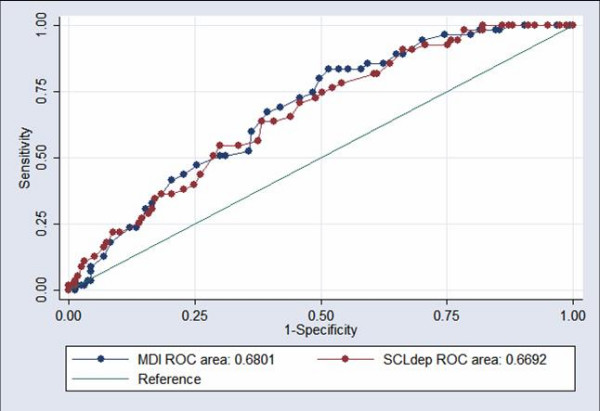
Receiver Operating Characteristic (ROC) analyses, with the MDI and the SCL-90-D, using the diagnosis of MDD as gold standard ^1)^. ^1) ^Mental disorders were assessed according to the DSM-IV criteria by experienced psychiatrists in a regular diagnostic interview.

We conducted ROC analyses for the MDI, and a separate ROC analysis in which we compared the MDI to the SCL-90 depression scale. The area under the curve was 0.68 for the MDI, which was about the same as the area under the curve for the SCL-D (0.67; non-significant difference; p = 0.73). The sensitivity, specificity, and percentage correctly classified is presented in Table 3, and the ROC curve is presented in Figure 1.

**Table 3:** Sensitivity (%) and specificity (%) of the Major Depression Inventory compared to DSM-diagnoses at different cut-off points (N = 228)

Cut-off	Sensitivity	Specificity
≥ 19	85.71	43.62
≥ 20	85.71	45.21
≥ 21	85.71	47.34
≥ 22	82.86	49.47
≥ 23	78.57	51.06
≥ 24	77.14	53.19
≥ 25	74.29	56.91
≥ 26	71.43	59.57
≥ 27	65.71	62.77
≥ 28	58.57	64.36
≥ 29	55.71	68.62
≥ 30	55.71	70.21
≥ 31	52.86	75.53
≥ 32	48.57	77.66
≥ 33	47.14	79.79
≥ 34	41.43	81.91
≥ 35	38.57	84.04

### The MDI as an indicator of MDD

We calculated the sensitivity and specificity of the indication of the presence of MDD based on the MDI (MDI-MDD) compared to the judgment of the psychiatrist (the gold standard). The sensitivity was 65.71, while the specificity was 64.89. We also calculated the kappa statistic as an indicator of the agreement between the two judgments. This was found to be 0.26, which according to the benchmarks from Landis and Koch [11] was moderate.

We compared the MDI-MDD in several subpopulations. In these analyses we selected the subjects with a specific diagnosis (while excluding the subjects with that diagnosis and a comorbid MDD), and compared these to the subjects with MDD (while excluding the subjects with MDD and the comorbid diagnosis). In this way, we compared subjects with an anxiety disorder (but no MDD) to subjects with MDD (but no anxiety disorder). The kappa of the MDI-MDD and the judgment of the psychiatrist was a moderate 0.25. In the same way, we compared subjects with MDD to subjects with dysthymia, with other mood disorder, with a bipolar disorder, with a psychotic disorder, with another neurotic disorder, with relational problems, with a substance use disorder, and with another disorder. In all cases, a moderate kappa (0.19–0.35) was found, indicating only a modest ability of the MDI to differentiate between subjects with MDD and other mental disorders in this population.

## Discussions and Conclusion

To our knowledge, this study is the first in which the psychometric characteristics of MDI are examined in different diagnostic categories of psychiatric outpatients. With a good reliability, a strong correlation with another measure of depressive symptomatology, and acceptable sensitivity and specificity, the MDI seems to be a useful and valuable new instrument for assessing depression in psychiatric outpatients.

One of the strong points of the MDI is that it can be used in two ways. First, it can be used as a continuous scale which indicates the level of depressive symptomatology. And second, it has an algorithm that allows clinicians to get an indication of the presence of MDD according to diagnostic criteria. However, our results show that the algorithm does not result in a higher sensitivity or specificity than a cut-off point on the MDI as a severity scale. This confirms the results of research in the general population, which also found that the MDI score rather than the MDI definition of major depression should be used in population based research [12].

In earlier reports on the MDI, a cut-off point of 26 was found for the MDI [2,12]. This cut-off point was supported by our study, with a sensitivity of 0.66, and a specificity of 0.63. This was much lower than in other studies of the MDI in clinical populations (with a sensitivity of 1.00 and a specificity of 0.82; [2]; and a sensitivity of 0.86 and a specificity of 0.94; [9]), but resembled the results of a study in the general population (sensitivity 0.61 and specificity 0.85, [12]).

This study has several limitations. First, no standardized instrument, such as the SCAN or the CIDI was used to assess the presence of mental disorders. In our study, the standard assessment by psychiatrists in routine practice was used as the gold standard. We did not assess inter rater reliability. This procedure may have resulted in an underestimation of the number of subjects with major depression, which in turn may have resulted in a reduced ability to differentiate subjects with major depression from those without. Second, most patients in our sample had more than one (Axis-I or Axis-II) disorder and this comorbidity may have distorted the results of our study. On the other hand, this high level of comorbidity reflects the actual complexity of patients seeking help in outpatient mental health care, and if this limits the results of the MDI, this should be seen as an indication that the MDI may not be used in this population. Third, the number of subjects in the different diagnostic categories was relatively small, which resulted in low power to detect differential effects. Because of these limitations, the results of this study have to be interpreted with caution.

The MDI is an attractive, brief depression inventory, which seems to be a reliable tool for assessing depression in psychiatric outpatients. More research with large samples and standardized diagnostic interviews is clearly warranted. Furthermore, an indication of the impairment criterion would most likely improve the specificity.

## Competing interests

The author(s) declare that they have no competing interests.

## Authors' contributions

PC conducted the analyses, and wrote the first draft and revisions of the manuscript. PC and JD conceived and planned the study. JD, AN and JP acquired the data. NS and JP completed the statistical analyses and data interpretation. All authors contributed to the drafting and revisions of the manuscript.

## Pre-publication history

The pre-publication history for this paper can be accessed here:


